# Enjoying art: an evolutionary perspective on the esthetic experience from emotion elicitors

**DOI:** 10.3389/fpsyg.2024.1341122

**Published:** 2024-02-26

**Authors:** Fabrizio Serrao, Alice Chirico, Alessandro Gabbiadini, Alberto Gallace, Andrea Gaggioli

**Affiliations:** ^1^Department of Psychology, University of Milano-Bicocca, Milan, Italy; ^2^Research Center in Communication Psychology, Universitá Cattolica del Sacro Cuore, Milan, Italy; ^3^IRCCS Istituto Auxologico Italiano, Milan, Italy

**Keywords:** beauty, esthetic experience, emotions, evolution, neuroesthetics, art

## Abstract

The ubiquity of human art prompted evolutionary psychologists to explore its origins as a potential adaptation to the environment. Here we focus on emotionally charged art and posit that affective affordances embedded into some artworks play a pivotal role in explaining why these artworks are enjoyed from an evolutionary perspective. Such features, recurring in various art forms, are interpreted as cues to the emotional state of others, enabling art consumers to engage in empathetic experiences and vicarious emotions. We explore the adaptive value of deriving pleasure from vicarious emotions, while also addressing the seemingly counterintuitive enjoyment of artworks that evoke negative emotions. We discuss the appreciation of vicarious emotions irrespective of their valence and maintain this appreciation to hold adaptive significance for three key reasons. Firstly, it aids art consumers in refining their interpretational schemes of internal states, potentially enhancing emotional regulation skills. Secondly, it contributes to a deeper understanding of the emotions of others, thereby fostering emotional intelligence and empathy. Lastly, the enjoyment of affectively charged artworks reinforces social cohesion by harmonizing the emotions of group members. This perspective provides a comprehensive framework for understanding the evolutionary underpinnings of the human capacity for art appreciation and emotional engagement.

## Introduction

*The emotions are sometimes so strong that I work without knowing it.*

*The strokes come like speech.*

*—*Vincent van Gogh—

Since the dawn of time, various human groups from all over the world have been producing what we consider art. Evolutionary biologists and anthropologists proposed that such a universal behavior was driven by environmental pressures, yet the ultimate causes of art production are still debated (Zahavi and Zahavi, 1997; Dissanayake, 2008; Prum, 2013). However, the borders between what is art and what is not are often blurred, making it hard to investigate this phenomenon with a scientific approach. In fact, the concept of art was often considered a social construct and was even argued to be absent in traditional societies (Dissanayake, 2008). Considering that the scope of experimental psychology is human experience, it seems more fruitful for psychologists and neuroscientists to focus on the impact of works of art on those who experience them. In many cases, the result of this interaction is esthetic pleasure, namely a release of opioids that is induced by stimuli in the visual and auditory domain (Blood and Zatorre, 2001).

The esthetic experience seems to depend on countless top-down factors—i.e., individual experience and knowledge, contextual cues, and socio-cultural aspects (Juslin, 2013; Chatterjee and Vartanian, 2014; Redies, 2015; McDermott et al., 2016; Pelowski et al., 2017; Verpooten and Dewitte, 2017; Van Geert and Wagemans, 2019). For example, the same artworks may elicit different emotions depending on autobiographical memories (Baumgartner, 1992; Janata et al., 2007; Pelowski et al., 2017; Jakubowski and Ghosh, 2021). Also, previous experience and expertise can even make art consumers dislike works of art that are widely appreciated (Juslin, 2013; Verpooten and Dewitte, 2017).

Nevertheless, it is remarkable that certain features recur in works of art from all cultures and times, such as symmetry and patterns (Gombrich, 1980; Westphal-Fitch and Fitch, 2018; Serrao, 2019). Several authors speculated that humans evolved to prefer specific features, which would thus elicit the esthetic experience in a bottom-up fashion—i.e., in a way that depends more on the form of the stimulus and less on the personal and cultural background of the art consumer. “Bio-aesthetics” (Westphal-Fitch and Fitch, 2018) is rooted in this assumption and seeks the ultimate causes of a cross-cultural preference for specific perceptual features in the ecology of our ancestors. Just like gustatory pleasure is a natural reward for eating foods that provide a selective advantage—e.g., foods rich in sugar or proteins,–esthetic pleasure might be a reward for some advantageous behavior.

For example, works of art displaying complex regularities like symmetric facades and geometric decorations are appreciated all over the world (Gombrich, 1980; Westphal-Fitch and Fitch, 2018; Serrao, 2019). Complex regular structures are made of different elements bearing logical-mathematical relationships with each other: the parts are diverse yet interconnected (Serrao, 2019). Thus, the ability to recognize complex regularity corresponds to the ability to spot connections between apparently unrelated elements, which is crucial for survival (Lakoff and Johnson, 2008). This ability enables to create parsimonious model for processing a varied set of stimuli, leading to saving processual energy (Friston, 2009). Alternatively, complex regular structures might be enjoyed because they can be processed more fluently, in line with the theory of perceptual fluency (Reber et al., 2004). According to this theory, features that facilitate the processing of a stimulus—such as symmetry, the goodness of form, and figure-ground contrast—would increase the enjoyment of that stimulus.

Other examples of cross-cultural esthetic preferences are those for colors (Yokosawa et al., 2010). Palmer and Schloss (2010) speculate that the most preferred colors—i.e., purple, and blue—are such because they characterize natural elements with a positive ecological valence—i.e., edible fruits and clear water ponds.

Yet another characteristic of widely appreciated works of art is to be emotionally stirring. It is noteworthy that people from different cultures are moved by works of art sharing similar features. Here, we focus on these features and discuss why they can trigger emotions in a bottom-up way. We maintain that humans evolved to perceive specific features in different sensory domains as affectively charged because such features are interpreted as cues of others’ emotions. The reason for this, in turn, could be that these features are generated through actions and behaviors that characterize a particular emotional state.

The contribution of emotions to the esthetic experience has been stressed by various authors (Konečni, 2008; Vuoskoski et al., 2012; Habibi and Damasio, 2014; Brattico et al., 2016; Etzi et al., 2016). Yet, while these authors effectively explain specific aspects or forms of art, none of them provides a unitary explanatory framework for the enjoyment of emotion-inducing art. Also, psychological models of the enjoyment of artworks describe *how* emotions are involved but often fail to explain *why* emotions became involved (see Juslin, 2013; Pelowski et al., 2017). In other words, previous authors discuss the near causes of enjoying being moved by artworks but overlook the ultimate causes of it, namely the reasons why our brains evolved in such a way. In this article, our goal is to merge and expand past proposals into a new framework capable of comprehensively elucidating the enjoyment of emotionally charged artworks across different sensory domains from an evolutionary perspective.

The proposed theoretical framework can also shed some light on a controversial matter: while the enjoyment of works of art that convey positive emotions may seem straightforward because of their inherent positive valence, the enjoyment of works of art conveying negative emotions is puzzling (Vuoskoski et al., 2012; Brattico et al., 2016). Why should we be willing to behold such a sorrowful sculpture as Michelangelo’s Pietà, or listen to such doleful music as Mozart’s Lacrymosa, or withstand the tribulations of Romeo and Juliet?

In an attempt to explain this paradox, some authors proposed a distinction between perceived emotions and felt emotions (Kivy, 1990; Kawakami et al., 2013). Accordingly, even if people deem a certain work of art to be an elicitor of negative emotions (i.e., perceived emotions), they will in fact report positive emotions (i.e., felt emotions) when asked about their personal experience with it. For example, Kawakami and colleagues show that listeners of sad music experience sadness to a lesser extent than expected and feel positive—i.e., romantic—emotions at the same time.

However, as Juslin (2013) points out, the paradox would persist: listeners did experience negative emotions. More importantly, the reason why art consumers would experience positive emotions from sad works of art remains unexplained. Pointing out that sad art elicits not only negative but also positive emotions does not solve the paradox: in fact, it only rephrases the paradox.

By the same token, Menninghaus et al. (2019) propose that art consumers experience a special kind of emotions—i.e., esthetic emotions—which do not necessarily coincide with the emotions represented in the artworks. Yet, evidence of distinct “aesthetic emotions” is inconsistent (Skov and Nadal, 2020).

Here, we address this conundrum by discussing the adaptive value of being moved irrespective of the emotional valence of the artwork—i.e., whether positive or negative. In line with the framework of “Bio-aesthetics” (Westphal-Fitch and Fitch, 2018), we argue that humans who enjoyed feeling emotion-inducing art could have had more chances to survive than those who did not. Therefore, it could be that these individuals learned to interpret the physiological changes in their bodies and the emotions of others with greater precision. Furthermore, they could feel more connected with each other and could cooperate with greater efficiency.

## Why is *Lacrymosa* sad and the *Egmont* awe-inspiring?

Even if the emotional load of a work of art depends on cultural and subjective factors, certain works of art are widely regarded as emotionally stirring. Take, for example, works of art depicting emotionally charged scenes or telling of emotionally charged events: it can be hypothesized that a maudlin story or a moaning portrait triggers sadness by means of empathy for the characters (Jahoda, 2005; Freedberg and Gallese, 2007). Similarly, while awe is often experienced when observing a vast open area from an elevated position—perhaps, as a positive reinforcement for being in control of the environment (Chirico and Yaden, 2018),—it can also be elicited by paintings of these views such as Friedrich’s Wanderer above the sea of fog^1^.

While the content of a work of art plays a key role in the affective charge of the work of art itself, “perceptual” characteristics can also trigger emotions across individuals and cultures (Blank et al., 1984). For example: the color of a visual stimulus (Wexner, 1954; Adams and Osgood, 1973; Valdez and Mehrabian, 1994; Palmer et al., 2013; Damiano et al., 2023), the pitch of an auditory stimulus (Juslin, 2000; Juslin and Laukka, 2003), or the phonemes of the letters in a text (Whissell, 1999; Aryani et al., 2016, 2018).

It has been proposed that perceptual features in a given sensory domain elicit a given emotion because they are interpreted as cues of that very emotion. That is, the body is more likely to produce those features during a specific emotional state (Juslin and Laukka, 2003; Freedberg and Gallese, 2007; Aryani et al., 2020). The primary function of an emotion is a response to the event that triggered it and thus it underlies physiological changes in the body that facilitate such a response (Scherer, 1985). For example, the physiological correlates of anger—e.g., muscle tension—prepare the body for aggressive behaviors. However, the underlying physiological changes would also impact the way signals are sent in different sensory domains, resulting in certain cues being associated with specific emotions. For example, muscle tension makes it more likely to vocalize hissing sounds and to draw broken lines, resulting in an association between these perceptual features and anger.

Accordingly, communication likely evolved from the initial ability to understand unintentional signals revealing the state of the sender (Bradbury and Vehrencamp, 1998). Subsequently, the sender would have learned to exploit this ability to send intentional signals.

In the case of artworks, they would be interpreted as signals that manifest the emotional state of others, eliciting the same emotions be the means of empathy (Freedberg and Gallese, 2007). Noteworthy, it is not necessary for the sender of the signal—i.e., the artist—to be known to the art consumer; in fact, the art consumer can empathize with a postulated sender by embodying the actions and behaviors through which the emotional cues are supposedly produced (Freedberg and Gallese, 2007).

On the other hand, artists might have empirically learned to leverage on emotion-inducing perceptual features for the purpose of expressing their emotions. Sometimes they exaggerate such features to elicit even greater emotional responses as a consequence of the peak shift effect (Ramachandran and Hirstein, 1999). Some artists tried to spell their empirical knowledge out loud. For example, the emotional force of lines and colors has been discussed by painter Kandinsky (2012).

In the following paragraphs, we address emotion-inducing features in different sensory domains and different forms of art.

### Emotion-inducing colors

According to Kandinsky, yellow (which he refers to as the “warmest” color) is joyful and keen, while blue (which he deems to be the “coolest” color) is soothing and restful. Indeed, early studies found that people associate yellow with cheerfulness and joy, while they associate blue with serenity and relaxation (Wexner, 1954). More recently, Damiano et al. (2023) found that anger is generally conveyed through the color red, fear is associated with black, sadness is linked to dark blue, and joy is expressed through yellow and pink. They also found sad drawings to be significantly less colorful than joyful drawings. Nevertheless, these studies did not investigate the specific contribution of each of the three parameters that define a color—i.e. hue, saturation, and brightness.

More in-depth studies found Kandinsky’s color theory to be only partially correct. Valdez and Mehrabian (1994) investigated how hue, saturation, and brightness correlate with emotions—the latter being described in terms of valence, arousal, and dominance. On the one hand, they found arousal to positively correlate with saturation; they also found it to correlate with brightness in a U-shaped fashion so that the least arousing colors are those with an intermediate degree of brightness.

Nevertheless, Valdez and Mehrabian (1994) found no significant correlation between hue and arousal if saturation and brightness are controlled for. Because the hue depends on the wavelength of color, these results do not support the common assumption that blue is more relaxing. A study by Palmer et al. (2013) found that bluish hues are rated as the calmest, yet the authors found no correlation between happiness and hue. Again, the latter finding challenges the widespread assumption that yellow is the most joyful among colors (Kandinsky, 2012). Perhaps, these assumptions stem from differences in brightness and saturation between those which are normally considered to be typical yellow (e.g., canary yellow) and typical blue (e.g., cobalt blue): the former differs from the latter not only in the hue but also in being more saturated (Valdez and Mehrabian, 1994). Indeed, Palmer et al. (2013) found happiness to positively correlate with saturation and brightness. This agrees with common sense: black—i.e., the dark shade par excellence—is normally considered the most somber color (Wexner, 1954). Also, the common belief that “warmer” colors (e.g., orange and yellow) are more arousing than “cooler” colors (e.g., blue and green) might stem from the tendency to consider more saturated and darker colors as warmer (Mehrabian and Russell, 1974).

Considering that brightness depends on the amount of light that is reflected by an object, the positive correlation between happiness and brightness may reflect that between mood and sunlight intensity. According to Kalbitzer et al. (2013), a decrease in sunlight is responsible for the so-called “winter depression:” it lowers the level of serotonin, which in turn has a negative impact on mood. Noteworthy, a reduction in serotonin not only affects mood but also triggers a set of behaviors that would be adaptive during harsh living conditions and food shortages (Kalbitzer et al., 2013). In other words, the negative effect of decreased sunlight on mood may be an evolutionary by-product of an adaptation to winter conditions (Kalbitzer et al., 2013).

Instead, the positive correlation between arousal and saturation accords with the widespread use of saturated colors as conspicuous signals by living organisms—from sexual ornaments to aposematic motifs, from floral blooms to mature fruits (Palmer and Schloss, 2010).

Among those associations between hue and emotions which are rooted in popular culture, only the one between anger and red withheld experimental testing. Indeed, Valdez and Mehrabian (1994) found red to be more arousing. Also, a correlation between redness and anger was confirmed by several studies (Palmer et al., 2013; Peromaa and Olkkonen, 2019; Damiano et al., 2023). At first glance, this correlation seems straightforward as red characterizes various aggressive behaviors, including the display of the teeth and gums, rage expressions, and facial redness (Humphrey, 1976; Peromaa and Olkkonen, 2019). This is confirmed by the finding that wearing red correlates with aggressiveness (Wiedemann et al., 2015) and that a red uniform can increase the chances of winning a fighting competition if there is no obvious ability gap between the opponents (Hill and Barton, 2005). However, it is worth noticing that other salient stimuli are also characterized by red and yet trigger completely different emotions: they range from open wounds to the genitals. Although it is unclear why the association between red and anger prevailed, it was found that men do not consider red female genitals more sexually arousing; in fact, pink genitals are preferred (Johns et al., 2012).

### Emotion-inducing lighting

Photographers know well that different emotional states can be elicited by playing with lighting. Specifically, “warmer” lighting is purported to elicit a sense of ease and comfort, while “colder” lighting elicits a sense of detachment and unpleasantness (Fraser and Banks, 2004). Such a popular assumption, which was confirmed in a cross-cultural study (Park and Farr, 2007), can be due to the apparent bluishness of dim light (Spitschan et al., 2017). This phenomenon likely results from rod-cone opponency in the retina (Joesch and Meister, 2016). A cinematographic technique called “day for night” showcases this as it enables to turn a day scene into a night scene by simply adding a blue filter. Because nighttime is characterized by a higher risk of being ambushed by predators and by lower temperatures, it might be the reason why “cooler” lights recall a state of discomfort.

Furthermore, blueish lighting was found to be more arousing than reddish lighting (Park and Farr, 2007). Blue light can also reduce sleepiness and increase attention both in the morning (Münch et al., 2016; Studer et al., 2019) and at night (Chellappa et al., 2011). This depends on the peak sensitivity in the blue waveband of melanopsin photoreceptors, which play a crucial role in the wake–sleep cycle regulation (Hankins et al., 2008). Perhaps, this peak sensitivity evolved because of the relative decrease in blue light by the end of the day (Chakraborti, 2007; Zagury, 2012). When the sun is lower in the sky, the path of the sunrays through the atmosphere is longer and the light is scattered by nitrogen to a greater extent: because shorter wavelengths—i.e., blue—are more scattered than longer wavelengths—i.e., red—blue light is extinguished and sunset sky acquires its typical reddish tinge (Chakraborti, 2007; Zagury, 2012).

Another purported effect of lighting has to do with the direction of the light. Specifically, when the light comes from below it may create a sense of disquiet (Millerson, 2013). The “hardness” of the shadows may also play an important role. That is, a sharp contrast between light and shadows, which normally occurs when the source of light is proximal, is said to be more intimidating than diffused light (Millerson, 2013). Both these unsettling lighting conditions are typically created by light sources that are lit up at night—such as bonfires,—while sunlight is more even. Again, this effect might relate to the threats of the nighttime such as predators and low temperatures.

### Emotion-inducing shapes

Visual features other than colors can also elicit specific emotions, as shown by experimental evidence (Blank et al., 1984; Takahashi, 1995). Specifically, Takahashi (1995) investigated the correlation between non-representational drawings that were meant to express specific emotions and the corresponding emotions. The author first enrolled artists to depict seven emotions using lines and shapes. He found that drawings representing the same emotions share several perceptual features. Noteworthy, the drawings remind of a series of artworks by Frida Kahlo titled Las Emociones, through which she depicted a variety of human emotions using lines, shapes, and colors.

Takahashi (1995) subsequently selected those drawings that were more expressive and asked participants to rate them on several dimensions. He also asked to rate the seven emotions on the same dimensions. He found that the rating of each drawing and the rating of the corresponding emotion did not differ.

Damiano et al. (2023) asked artists and individuals without artistic backgrounds to represent a set of emotions—i.e., anger, disgust, fear, sadness, joy, and wonder—using abstract drawings. They found that drawings meant to convey sadness featured significantly more vertical lines compared to all other drawings. Similarly, when discussing the effect of the direction of lines, Kandinsky concludes that lines going upwards convey positive emotions, while lines going downwards convey negative emotions (even though the direction probably depends on contextual factors, such as the reading direction of a specific culture).

It is plausible that certain forms are associated with specific emotions because they are interpreted as cues of those emotions: the movements required to produce those forms are more likely when a specific emotion is ongoing (Freedberg and Gallese, 2007). For example, muscle tension characterizes anger and is likely to result in drawing broken lines, leading to the association between anger and broken lines. This is consistent with the emotional mediation hypothesis for explaining the “bouba-kiki” effect (Aryani et al., 2020). Namely, people would cross-culturally match the sound “kiki” with a spiky figure because they associate both stimuli with the same emotion. This line of thought also applies to Pollock’s “action painting”^2^ and Fontana’s cut canvases^3^, and even to architectural elements like bent or twisted structures (Freedberg and Gallese, 2007).

Sometimes, certain forms might result in the art consumer empathizing with the work of art itself. Such forms resemble the way in which certain emotions are expressed through the body. For example, Blank et al. (1984) argue that lines going downwards are considered sad because they remind of the sagging posture and facial expression characterizing sadness.

### Emotion-inducing auditory cues

The coupling of dissonant tones, which is experienced as “roughness” (von Helmholtz, 1863), was found to trigger defensive responses and negative emotions (Di Stefano and Spence, 2022). The notion of consonance and dissonance is key in composition and can be considered as one of the most salient properties of Western music (Di Stefano et al., 2022).

Besides, Juslin (2000) reviewed several studies employing the “standard context paradigm” (Davitz, 1964) to elucidate how emotions are conveyed by auditory cues other than the melody. In these studies, musicians were asked to convey different emotions through the same melody, and listeners were asked to identify such emotions. Juslin concludes that musicians consistently express emotions by modulating the loudness, the pitch, the tempo, and the articulation of a piece of music. The author also concludes that listeners use the same auditory cues to understand what emotions are expressed by musicians. For example, emotions with a higher arousal level are conveyed through higher sound levels, faster tempo, faster tone attack, and more variability compared to emotions with a lower arousal level. This seems to apply to different cultural backgrounds. In other words, certain features of music seem to be inherently emotion-inducing (Juslin, 2000, 2013; Juslin and Laukka, 2003).

Hailstone et al. (2009) found that the timbre of different musical instruments can convey different emotions when the melody is controlled for. In their study, the same melody intended to convey one of four target emotions—happiness, sadness, joy, anger—was played with different instruments—piano, violin, trumpet, and synthesizer—and participants were asked to guess the target emotion. They found that happy melodies were less likely to be identified when played by violin, sad melodies were less likely to be identified when played by synthesizer, and angry melodies were less likely to be identified when played by trumpet (Hailstone et al., 2009).

In this regard, it has also been proposed that music can borrow emotion-inducing cues from vocal communication (Juslin and Laukka, 2003; Di Stefano, 2023). Indeed, there is a correspondence between the cues through which the same emotions are conveyed in music and vocal communication. For example, the tempo of music corresponds to the speech rate and they produce similar emotional effects (Juslin, 2000). Also, “rough” timbres and vocalization in stressful situations—e.g., screams—have similar spectral qualities (Di Stefano and Spence, 2022; Di Stefano, 2023).

Juslin and Laukka (2003) propose that the emotional load of certain cues in vocal expression—and, consequently, of corresponding cues in music—relates to the physiological correlate of emotions, which consists of changes in respiration rate, muscle tension, and vocal folds vibration. For example, anger is characterized by increased laryngeal muscle tension and subglottal air pressure. These changes, in turn, affect sound production and make certain sounds more likely to be produced than others. In other words, humans evolved to match auditory cues with a specific emotion because such cues reveal the emotional state of the sender.

Nevertheless, because different emotions may underlie similar physiological correlates, auditory cues are interpreted in a probabilistic way (Juslin, 2000, 2013; Juslin and Laukka, 2003). For example, sadness and tenderness seem to share many auditory cues and thus they were frequently confused by listeners (Juslin, 2000). This observation stresses the importance of contextual cues—which, in the case of a piece of music, could be the lyrics—in discerning emotions. The emotional power of music might also result from cross-modal associations (Di Stefano, 2023). That is, certain musical features might convey a certain emotion because they mirror behaviors typical of that emotion. For example, a slow tempo may convey sadness because it recalls the slow movements of a sad person.

Finally, some auditory cues might elicit a specific emotion because they resemble the sound of natural phenomena. For example, sublime pieces of music—like Beethoven’s Egmont—might recall the sound of a storm. Indeed, the experience of the sublime arises, according to Kant (1914), when observing a fearful phenomenon from a safe position (Kant, 1914; Gordon et al., 2017). Accordingly, Vuust and Kringelbach (2010) propose that chills experienced by music listeners are a frightened response to a sudden and unpredictable change in the structure of the piece of music—e.g., when a new voice joins in.

### Emotion-inducing phonemes

The use of specific phonemes in literature seems to correlate with the expression of specific emotions (Whissell, 1999; Aryani et al., 2016, 2018). Again, such phonemes are more likely to be produced during a certain emotional state because of the physiological correlate of that emotion. This is in line with the vocal theory of language, according to which language evolved from instinctive calls that were made in specific situations. Noteworthy, the emotional load of the letters is retained even when they are read silently (Aryani et al., 2016, 2018).

More specifically, Aryani et al. (2016) found a negative correlation between the length of vowels and arousal in German poems. They point out that shorter vowels are produced through a briefer release of air compared to long vowels, which resembles fast breathing at high arousal. Also, the authors found that voiceless, plosive, and hissing consonants are more arousing than their counterparts. The articulation of such sounds, which requires greater muscular force and oral pressure, coheres with the physiological correlates of emotion with a higher arousal level. Accordingly, Auracher et al. (2010) point out that plosive sounds are frequent in poems expressing happiness in German, Portuguese and even Ancient Egyptian.

Instead, “l” seems to be more frequent in English, French, and Hungarian texts expressing tenderness, perhaps because facial muscles are relatively relaxed when articulating this sound (Whissell, 1999).

Noteworthy, animals like birds and non-human primates also use harsh sounds for hostile calls and tone-like sounds for friendly calls (Juslin and Laukka, 2003; Aryani et al., 2018; Di Stefano and Spence, 2022).

Some phonemes are articulated through a configuration of facial muscles resembling the facial expression of emotions. This could be another reason why some letters are emotionally connotated. For example, the higher frequency of “e”—as in “see”—in happy English texts can be due to the way it is pronounced; namely, with a smile-like contraction of facial muscles (Whissell, 1999). On the contrary, the “ɑ” sound—as in “llama”—is pronounced with a sagging mouth resembling the expression of sadness. Similarly, the articulation of nasal consonants like “n,” which requires keeping the mouth closed, simulates a serious expression. Indeed, both “ɑ” and “n” seem to be more common in sad texts (Whissell, 1999; Auracher et al., 2010).

### Emotion-inducing textures

The role of the sense of touch in the experience of art might not seem so obvious (Gallace and Spence, 2014). Yet, futurist Martinetti even proclaimed the birth of an artistic movement revolving around touch, namely Tattilismo. In one of his artworks, titled Sudan-Parigi (meaning “Sudan-Paris” in Italian), Marinetti made use of different textures—such as sandpaper and velvet—to evoke these two locations through tactile experiences.

Although touch is not so commonly used to express emotions in art, Etzi et al. (2016) found that different textures are associated with different emotions. Specifically, positive emotions are associated with smoother textures like tinfoil and satin. According to the authors, this association stems from smooth textures having a positive valence just like positive emotions do. In turn, the positive valence of smooth surfaces relates to the stimulation of C tactile fibers, which also mediate the pleasantness of being tenderly stroked (Etzi et al., 2018).

## Evolution rewards the sensitive

The enjoyment of emotion-lad artworks poses a theoretical conundrum. It can be said that positive emotions are rewarding because of their inherent positive valence, yet the negative valence of negative emotions theoretically leads to avoiding stimuli that induce them (Damasio, 1994). Nevertheless, works of art charged with negative emotions are widely enjoyed (Vuoskoski et al., 2012; Brattico et al., 2016). We build upon the idea that enjoying emotionally stirring works of art is adaptive regardless of the emotional valence because the emotions are felt vicariously (Yılmaz et al., 2019). That is, the emotion is caused by a stimulus that does not require the behavioral response for which that emotion evolved. For example, a horror movie can elicit fear, whose physiological correlate prepares for a fight-or-flight response, even though there is no need to escape the movie scene. In the following paragraphs, we elaborate on the adaptive value of experiencing vicarious emotions.

### Vicarious emotions refine the understanding of one’s own emotions

The vicarious experience of emotions may train to make sense of one’s own emotions. This is consistent with the view, dating back to James’ theory (James, 1884), that emotions arise as a cognitive integration of the internal state of the body with situational cues. The brain would end up categorizing an emotion as such by interpreting physiological changes—which are often shared by different emotions—in light of past experience and conceptual knowledge (Barrett, 2006). Indeed, scientific evidence does not support the view of emotions as discrete neurobiological entities (Barrett, 2006).

Accordingly, Damasio and Carvalho (2013) draw the difference between emotions and feelings. They propose that the former are physiological changes while the latter are the conscious interpretation of such changes. They argue that:

“Directly portraying the advantageous or disadvantageous nature of a physiological situation as a “felt experience” facilitates learning of the conditions responsible for homeostatic imbalances and of their respective correlation, as well as anticipation of future adverse or favorable conditions” (Damasio and Carvalho, 2013, p.1).

Moreover, Damasio (1994) postulates that the brain constantly interprets physiological changes in the body and “marks” the outcome of the actions accordingly. These emotional “markers” are crucial for making decisions efficiently.

The conception of emotions as cognitive interpretations of physiological changes is supported by findings that people are highly reliant on contextual cues when categorizing emotions (Trope, 1986). Also, the act of mimicking facial expressions of a specific emotion was found to induce the feeling of that emotion—e.g., forcing a smile can make one feel happier (Ekman et al., 1983; Whissell, 1985). In other words, those bodily reactions that are generally considered to be induced by emotions might in fact cause the feeling of an emotion.

Also, the context can lead the brain to mis-categorize physiological changes. For example, it was found that participants who interacted with a good-looking interviewer in a dangerous scenario were more likely to report feelings for the interviewer (Dutton and Aron, 1974). These results led the authors to conclude that participants mistook the physiological cues of fear—higher heartbeat etc.—with those of infatuation.

In light of this, we hypothesize that experiencing vicarious emotions positively impacts the ability to interpret the physiological changes in the body. Such an interpretation corresponds to the very feeling of an emotion. This idea fits well within the predictive coding framework (Friston, 2009), which postulates that the brain constantly explains sensory inputs—including those about body states—by comparing them with *a priori* models. A mismatch between the two—i.e., a “prediction error”—would require further elaboration and thus a greater energy expanse. By refining its model of the physiological changes occurring in the body, the brain would make better predictions on the changes themselves and minimize prediction errors. In other words, works of art can be considered a means of social learning, which is based on the observation of others’ behavior (Grusec, 1994; Yılmaz et al., 2019).

An increased experience with emotions from vicarious emotions would result in feeling emotions with finer granularity. In support of this hypothesis, it was found that arts, music, and poetry help students develop their emotional intelligence (Morris et al., 2005; Kumschick et al., 2014; Clarke et al., 2016). This, in turn, enables one to deal with their own internal states in a better way and to communicate emotions with greater precision (Barrett et al., 2001; Barrett, 2006; Kumschick et al., 2014). Accordingly, Barrett et al. (2001) found that people who can better differentiate their own negative emotions can also perform emotional regulation more efficiently.

Noteworthy, gaining additional experience with one’s own internal states is even more advantageous in the case of negative emotions. By feeling negative emotions vicariously, art consumers can gain experience with such emotions in a safe environment, namely when their survival is not at risk. Let us use a metaphor: vicarious emotions provide an opportunity to learn about emotions in a safe context just like visiting the aquarium enables one to observe sharks with no risk of being attacked.

### Vicarious emotions refine the understanding of others’ emotions

A finer categorization of one’s own emotions may result in a better ability to infer the internal state of others and thus respond more efficiently. In other words, it may foster emotional intelligence, affective theory of mind, and empathy (Barrett, 2006; Konečni, 2008; Kidd and Castano, 2013). Emotion-inducing artworks can showcase how it feels in a multiplicity of scenarios, including scenarios that the art consumer might never encounter directly. Needless to say, the ability to understand each other’s behavior is crucial for social interactions.

In support of this hypothesis, Kumschick et al. (2014) found that a literature-based program fosters students’ ability to understand the emotions of others. Kidd and Castano (2013) found that readers of novels are more empathetic than non-readers and readers of non-fictional books. Similar results were obtained by Mar et al. (2006), while Hakemulder (2000) found that participants who read fictional texts about women’s condition in fundamentalist Islamic countries were able to empathize with women in such countries more than participants who read non-fictional texts on the same topic.

Noteworthy, literary fiction compels readers to engage in mind-reading to understand multifaced characters, fill narrative gaps, and find implicit meanings (Kidd and Castano, 2013). Moreover, in the absence of a singular authorial perspective, they are prompted to embrace multiple viewpoints (Kidd and Castano, 2013).

Concerning other fields of art, medical students reported that discussing visual works of art enhanced their ability to feel the suffering of others (Bentwich and Gilbey, 2017), while nursing students who visited an art gallery reported that the experience increased their understanding of interpersonal relationships in nursing situations (Wikström, 2000). Also, Greene et al. (2014) found that school students who went on a trip to an art museum could better empathize with the people who lived in the past. A comprehensive survey by Kou et al. (2020) found that visual and performing arts enthusiasts report higher empathetic concern for others. In line with all these findings, the experience of art activates the same areas of the cerebellum—e.g., lobule VII—that are involved in perspective-taking and empathy (Adamaszek et al., 2022).

### Shared emotions increase group cohesion

Emotion-lad works of art might be instrumental in promoting social bonding (Bouriaud, 2002; Juslin and Laukka, 2003; Redies, 2015). Art consumers feel deeply interconnected when an artwork stirs the same emotion across them. This phenomenon might be particularly important for increasing in-group cohesion, as in the case of folk songs and patriotic poems (Juslin and Laukka, 2003; Redies, 2015). According to Wilson (2000), the father of Sociobiology, music evolved to tune the emotions of the members so that the group can jointly pursue a common objective. This can also apply to all other forms of art. Take, for example, depictions of triumphant armies or of the sacrifice of a hero, or even Christian representations of the tribulation of Jesus.

Accordingly, Dunbar et al. (2016) found that people who watched an emotionally stirring movie in groups eventually felt more bound to each other compared to those who watched emotionally neutral movies. Moreover, art therapy, which aims at building relationships between individuals through art, can increase mutual understanding and trust; it is believed to induce the secretion of oxytocin (Springham et al., 2014; Springham and Huet, 2018). More generally, shared emotions can foster group bonding and result in better collective performance (Kelly et al., 2014).

It is worth mentioning that music and dancing, whether or not emotionally stirring, are believed to have evolved to promote group cohesion (Hattori, 2021; Savage et al., 2021). Perhaps, the emotional power of music adds to this effect and further cements groups.

## Conclusion and future research directions

Here, we reviewed evidence suggesting that certain perceptual features of an artwork can be inherently moving. Indeed, such features seem to recur in emotionally charged artworks. While previous authors who discussed this matter focused on specific sensory domains, we maintain that the same mechanism underlies the enjoyment of emotion-inducing perceptual features across sensory domains. We propose a general framework for understanding how perceptual features can arouse emotions in all arts—from literature to music to visual arts. That is, the above-listed features of artworks are inherently moving because they are interpreted as cues of the emotional states of others. Consequently, they make the art consumers feel the same emotions by means of empathy.

In addition, we maintain that humans evolved to enjoy emotion inducing works of art. The enjoyment of objects that trigger positive emotions is straightforward because of their positive valence, yet the enjoyment of objects that elicit negative emotions is apparently counterintuitive. We attempt to solve this paradox by proposing that experiencing emotions vicariously through artworks is adaptive regardless of their valence. Specifically, the vicarious experience of emotions trains art consumers to interpret the physiological changes in their body—which corresponds to the very experience of emotions (Barrett et al., 2001; Barrett, 2006)—and the emotional state of others. Furthermore, we argue that emotion-inducing artworks reinforce the in-group cohesion by tuning the emotions of its members.

Three clarifications are needed. First, we discussed why it is adaptive to experience emotions vicariously through works of art, yet art is not the only way to experience so. For example, sports fans can vicariously experience pride and shame as a consequence of the performance of their team (Sullivan, 2014; Partridge et al., 2020). Yet, while these vicarious emotions can also increase group bonding (Kelly et al., 2014), art can perhaps foster emotional intelligence more effectively because of its more complex and nuanced emotional repertoire. However, it is worth noting that the scope of the present article is the enjoyment of art and therefore we were interested in discussing vicarious emotions in this context only.

Second, while we discussed the ultimate causes of enjoying emotionally stirring artworks, we did not address the drives to produce such works of art. It can be speculated that experiencing vicarious emotions and expressing emotions have similar adaptive purposes. That is, some artists might be driven to express their emotions because it enhances their ability to understand their own internal states (Springham and Huet, 2018) and that of others (Kou et al., 2020). It might even make them feel connected to consumers of their artworks (Gonzales et al., 2010; Springham et al., 2014).

Alternatively, some artists might seek to create emotional-lad and thus enjoyable manufacts for the purpose of social acknowledgement. According to the sensory trap hypothesis (Christy, 1995), an attraction for a specific stimulus may even predate the ability to produce that stimulus. In any case, while the adaptive value of producing emotion inducing artworks might be intertwined with the adaptive value of experiencing such artworks, the former remains a distinct matter and is beyond the scope of the present article.

Third, the fact that we discuss the enjoyment of art does not necessarily imply that art evolved to be liked. In fact, art served countless purposes throughout the history of humankind other than pleasing the senses. For example, art was a form of propaganda and was instrumental in perpetuating the memory of important events when most people could not read. In any case, the purpose of art is beyond the scope of this article.

By highlighting that the consumption of affectively charged artworks is adaptive, we also imply that such artworks can have a positive impact on people’s lives. For example, the experience of art can be instrumental in the emotional education of young individuals, enabling them to better understand themselves and others. Moreover, the cohesive effect of shared intense experiences through art might serve the purpose of increasing cooperation within teams or communities. Experimental evidence of these effects is promising yet sparse and context-specific: further data are needed to draw more general conclusions. Still, we expect policies that facilitate the incorporation of art into our daily lives to prove tremendously beneficial and we encourage policymakers and public institutions to implement them. This will help to give meaning to van Gogh’s statements and turn art into a beneficial tool for educating our emotions.

## Data availability statement

The original contributions presented in the study are included in the article/supplementary material, further inquiries can be directed to the corresponding author.

## Author contributions

FS: Conceptualization, Writing – original draft, Writing – review & editing. AC: Writing – review & editing, Supervision. AGab: Writing – review & editing, Supervision. AGal: Writing – review & editing, Supervision. AGag: Writing – review & editing, Supervision.

## Footnotes

^1^https://www.wikiart.org/en/caspar-david-friedrich/the-wanderer-above-the-sea-of-fog

^2^https://www.wikiart.org/en/jackson-pollock/number-5-1948-1

^3^https://www.wikiart.org/en/lucio-fontana/concetto-spaziale-1964
